# Editorial: New Advances in Identification and Quantification of Foodborne Pathogens

**DOI:** 10.3389/fmicb.2021.783406

**Published:** 2021-11-01

**Authors:** Dario De Medici, David Rodriguez-Lazaro, Nigel Cook

**Affiliations:** ^1^Istituto Superiore di Sanità, Rome, Italy; ^2^Microbiology Division, Faculty of Sciences, University of Burgos, Burgos, Spain; ^3^Centre for Emerging Pathogens and Global Health, University of Burgos, Burgos, Spain; ^4^Jorvik Food and Environmental Virology Ltd., York, United Kingdom

**Keywords:** food safety, molecular methods, rapid method development, foodborne pathogen detection, foodborne virus concentration, next generation (deep) sequencing (NGS), droplet PCR

Microbiological analysis is an integral part of food safety quality control and monitoring systems. Recent outbreaks, associated with consumption of a variety of contaminated foods, have highlighted the difficulty in monitoring critical points of contamination along the food chain. The rapid and precise detection (and/or quantification) of foodborne pathogens, used appropriately along the food supply chain, represents a powerful tool to prevent outbreaks of foodborne disease. With this purpose in mind, and particularly for short shelf-life foods, it is necessary to increase the arsenal of available methods and develop innovative and time-effective assays which are able to complete the analytical process in as short time as possible. In this context, the major benefit of molecular methods, as alternatives to classical culture-based methods, is the significant reduction of the time taken for analysis. However, basic molecular approaches have also some critical weaknesses, such as an inability to discriminate between viable and dead cells (or infectious and non-infectious virus particles), and susceptibility to inhibitory influences.

In recent years, there have been several advances in molecular-based subtyping methods, particularly regarding the application of sequence-based techniques. Different molecular methods, including more recently next- generation-based approaches, are replacing traditional methods in identifying the source of outbreaks, improving trace-back studies, and elucidating the evolution of foodborne pathogens. However, all these techniques need further validation before being adopted internationally.

This Research Topic encompasses different aspects regarding the application of molecular methods for the detection, quantification, and identification of foodborne microorganisms, including the proposition of innovative solutions. The Special Issue comprises 12 original articles, contributed by 111 authors from 14 different countries.

## Specific Detection of Viable/Infectious From Dead/Non-Infectious Targets

The major challenge of molecular methods is their inability to differentiate between dead/infectious and viable cells/non-infectious microorganisms (Zeng et al., 2016). An approach to address this is proposed within this issue.

Zhang et al. optimized a commercial extraction kit, PMAxx-based qPCR for the quantification of viable *Salmonella* Enteritidis and *Salmonella* Typhimurium from poultry-production environments. The method was successfully optimized and applied to assess the survivability of *Salmonella* serovars in soil samples, incubated at 3 different temperatures (5, 25, and 37°C) over 6 weeks.

A very different approach is proposed by Xu et al., which utilizes pathogen interaction with host cells to detect only live microorganisms. They exploited the ability of viable enteric pathogens to adhere to intestinal cells, harnessing an antibody-based assay for specific detection of the adhered target pathogens. The Authors also proposed an innovative approach to prolong the shelf-life of cells (for at least 14 weeks) for possible point-of-need deployment and multi-sample testing on a single plate.

## Advance in Extraction Methods for Enteric Viruses

Detection of enteric viruses in food products differs from detection of most foodborne bacteria, as viruses cannot be enriched in a food sample. The pre-PCR processing step, including sample preparation and nucleic acid extraction, is a critical step in foodborne virus detection. The quality of the extracted nucleic acids can strongly affect the efficiency of detection by a molecular method. In 2018 an outbreak of hepatitis A, caused by contaminated dates, was identified in Denmark by Rajiuddin et al. A new direct lysis method for the extraction of viral RNA from dates was developed and optimized to detect the virus, quantify the contamination level, and sequence the strain(s) detected.

## Same Day Detection Methods

The length of the enrichment time is a bottleneck for development of culture-based methods able to detect bacterial pathogens in the same day (Fachmann et al., 2017).

Garrido-Maestu et al. applied a rapid short enrichment step followed by an innovative unique multiplex real time PCR method, to mediate same-day detection and confirmation of *Salmonella* spp and *E. coli* O157. The method was also validated for stressed and dead cells inoculated into meat, demonstrating that it is capable of distinguishing thermally stressed bacteria, as well as avoiding false positive results due to the presence of dead bacteria.

Li et al. propose a multiplex PCR (mPCR) for rapid identification of *Listeria monocytogenes* and *Listeria ivanovii*, and non-pathogenic *Listeria* in edible mushroom (*Flammulina velutipes*) samples after a short enrichment culture step (4–12 h).

## New Molecular Approach: Absolute Quantification and Next Generation Sequencing

Real time PCR or qPCR has been widely used for the quantification of nucleic acids with high precision, but it can be inaccurate when targets are at very low concentrations. Droplet digital polymerase chain reaction (ddPCR), a relatively new and promising technology, has many advantages over qPCR, as it can provide greater sensitivity and accuracy. It can be used for ultrasensitive and absolute nucleic acid quantification without a standard curve (Peng et al., 2020).

Villamil et al. (2020) validated a method based on the application of a simplex and duplex droplet digital polymerase chain reaction (ddPCR)-based method, for the identification and quantification of *Salmonella*. The Authors demonstrated that the use of a multiplex detection, targetting *ttr, inv*A, *hil*A, *spa*Q, and siiA genes, provides more reliable quantification, particularly for some specific applications such as reference material characterization.

Recently, Next Generation Sequencing has become a powerful tool for epidemiological investigation, as it can define the causative agents of disease, trace the origin of the pathogen, and clarify routes of transmission. Within the framework of the European Union-funded project COMPARE [Collaborative Management Platform for detection and Analyses of (Re-) emerging and foodborne outbreaks in Europe], Hoper et al. present the results of a proficiency test to scrutinize the ability of 12 different laboratories to assess diagnostic metagenomics data, based on the use of an artificial dataset resembling shotgun sequencing of RNA from a sample of contaminated trout. Analysis of the results demonstrate that a reliable classification of the reads can be obtained by the software used.

The consistency, accuracy and reproducibility of next-generation short read sequencing, between different laboratories and platforms, has been evaluated by Uelze et al. The Authors report the results of a ring trial performed in private and public laboratories, sequencing DNA samples of three bacterial species (*Campylobacter jejuni, Listeria monocytogenes*, and *Salmonella enterica*), according to their routine in-house sequencing protocols. Four different types of Illumina and one Ion Torrent sequencing platform in ten different laboratories were involved in the study. The results show differences in data quality after assembling short reads into genome assemblies.

The bioinformatics pipelines of whole genome sequencing (WGS) data, generated by the long-read sequencing platform Oxford Nanopore Technologies (ONT), have been evaluated by Wu et al. Five isolates of *Salmonella* were analyzed, and the results demonstrated the reduction of the cost of ONT sequencing of a single isolate per flow cell, the achievement of high coverage of the genome, and a high accuracy of prediction within 1 day. This study provides food industries, food authorities, and regulators with the alternative of short-read sequencing platforms for serotype prediction for pathogen surveillance.

## Immunodiagnostic Assay

The major economic threat to the marine aquaculture industry, with regard to consumer health, is the presence of *Vibrio* species in seafood.

Development of an accurate immunodiagnostic assay of pathogenic *Vibrio* species, using highly specific unique outer membrane proteins (OMPs), is described by Wang et al. The Authors have applied a bioinformatic tools for screening OMPs candidates, to minimize target numbers in “*in vivo*” tests.

## Monitoring

The Research Topic also includes two extensive surveillance studies in which the Authors apply molecular methods for characterizing the isolated strains. Very importantly, both papers have a particular focus on antibiotic-resistant bacteria (Aijuka and Buys, 2020).

The paper presented by Li et al. reports the results of a survey in which new molecular technologies have been applied for evaluating *Salmonella* presence in eggs in a specific region of China, with extensive characterization of isolates. The results of the work indicate that more attention should be paid to egg production, transportation, and storage to prevent foodborne outbreaks caused by *Salmonella*.

Finally, an extensive investigation of the prevalence of methicillin-resistant *Staphylococcus aureus* (MRSA) in slaughterhouses and meat shops in Pakistan has been presented by Sadiq et al. The authors collected a total of 300 samples. Fifty per cent of the analyzed samples were positive for *S. aureus* by phenotypic identification, and 96 isolates (63%) showed resistance to the antibiotic cefoxitin, known as a potential marker for detecting MRSA. All 150 isolates showed complete resistance to four antibiotics–neomycin, methicillin, ciprofloxacin, and tetracycline.

## Author Contributions

All authors listed have made a substantial, direct and intellectual contribution to the work, and approved it for publication.

## Conflict of Interest

The authors declare that the research was conducted in the absence of any commercial or financial relationships that could be construed as a potential conflict of interest.

## Publisher's Note

All claims expressed in this article are solely those of the authors and do not necessarily represent those of their affiliated organizations, or those of the publisher, the editors and the reviewers. Any product that may be evaluated in this article, or claim that may be made by its manufacturer, is not guaranteed or endorsed by the publisher.

## References

[B1] Aijuka M. Buys E. M. (2020). Detection of extended-spectrum beta-lactamase cefotaxime resistance and virulence genes in Escherichia coli by duplex quantitative real-time PCR and melt curve analysis. Lett. Appl. Microbiol. 71, 54–60. 10.1111/lam.13274 31930506

[B2] Fachmann M. S. R. Löfström C. Hoorfar J. Hansen F. Christensen J. Mansdal S. Josefsen M. H. (2017). Detection of *Salmonella* enterica in meat in less than 5 hours by a low-cost and noncomplex sample preparation method. Appl. Environ. Microbiol. 83, e03151–e03116. 10.1128/AEM.03151-16 27986726 PMC5311390

[B3] Peng C. Zheng M. Ding L. Chen X. Wang X. Feng X.. (2020). Accurate detection and evaluation of the gene-editing frequency in plants using droplet digital PCR. Front. Plant Sci. 14:610790. 10.3389/fpls.2020.610790 33381141 PMC7767858

[B4] Villamil C. Calderon M. N. Arias M. M. Leguizamon J. E. (2020). Validation of droplet digital polymerase chain reaction for *Salmonella* spp quantification. Front. Microbiol. 11:1512. 10.3389/fmicb.2020.01512 32733415 PMC7358645

[B5] Zeng D. Chen Z. Jiang Y. Xue F. Li B. (2016). Advances and challenges in viability detection of foodborne pathogens. Front. Microbiol. 7:1833. 10.3389/fmicb.2016.01833 27920757 PMC5118415

